# Foxk2 Enhances Adipogenic Differentiation by Relying on the Transcriptional Activation of Peroxisome Proliferator‐Activated Receptor Gamma

**DOI:** 10.1111/jcmm.70332

**Published:** 2025-01-09

**Authors:** Shan Zhang, Yanru You, Ran Li, Mingcong Li, Yachong Li, Hairui Yuan, Jie Zhou, Ruonan Zhen, Ying Liu, Baoli Wang, Endong Zhu

**Affiliations:** ^1^ NHC Key Lab of Hormones and Development and Tianjin Key Lab of Metabolic Diseases Tianjin Medical University Chu Hsien‐I Memorial Hospital & Institute of Endocrinology Tianjin China; ^2^ Department of Endodontics, School of Stomatology Hospital of Stomatology, Tianjin Medical University Tianjin China; ^3^ Hebei International Travel Healthcare Center (Shijiazhuang Customs Port Clinic) Shijiazhuang Hebei province China

**Keywords:** adipocyte, BMSC, differentiation, Foxk2, PPARγ

## Abstract

Proper differentiation of bone marrow stromal cells (BMSCs) into adipocytes is crucial for maintaining skeletal homeostasis. However, the underlying regulatory mechanisms remain incompletely understood, posing a challenge for the treatment of age‐related osteopenia and osteoporosis. Here, through comprehensive gene expression analysis during BMSC differentiation into adipocytes, we identified the forkhead transcription factor Foxk2 as a key regulator of this process. Foxk2 expression was significantly higher in the inguinal and epididymal white adipose tissues of db/db mice compared to non‐obese db/m controls and was induced in BMSCs, C3H/10 T1/2, and ST2 cells following adipogenic stimulation. Overexpression of Foxk2 promoted adipogenic differentiation of C3H/10 T1/2, ST2, and BMSCs, accompanied by increased expression of lipogenic factors. Conversely, Foxk2 silencing inhibited adipogenic differentiation. Moreover, Foxk2 also facilitated lipogenesis of C3H/10 T1/2 and ST2 cells. Adipogenic stimuli triggered the nuclear translocation of Foxk2 through PI3‐kinase and mTOR signalling pathways. Once in the nucleus, Foxk2 is directly bound to the promoters of Pparγ1 and Pparγ2, thereby enhancing their transcriptional activity. Notably, PPARγ1 and PPARγ2 reciprocally augmented the transcriptional activity of the Foxk2 promoter, indicating the presence of a Foxk2‐PPARγ positive feedback loop that drives adipogenesis.

**Abbreviations**
Acly: adenosine triphosphate citrate lyase
AIM: adipocyte‐inducing medium
BAT: brown adipose tissue
BMAds: bone marrow adipocytes
BMAT: bone marrow adipose tissue
BMSCs: bone marrow stromal cells
C/EBP: CCAAT/enhancer binding protein
ChIP: chromatin immunoprecipitation
Fabp4: fatty acid binding protein 4
Fasn: fatty acid synthase
FBS: fetal bovine serum
Foxk: forkhead box k
Fsp27: 27‐kDa fat‐specific protein
PPARγ: peroxisome proliferator‐activated receptor γ
RT‐qPCR: reverse transcription‐quantitative polymerase chain reaction
SREBP: sterol regulatory element‐binding protein
TF: transcription factor
WAT: white adipose tissue

## 1 Introduction

Bone marrow adipocytes (BMAds) reside within the bone cavity alongside haematopoietic cells, trabecular bone, nerve fibres, blood vessels and sinusoidal capillaries, which originate from bone marrow mesenchymal stromal cells (BMSCs) [1, 2]. These BMSCs not only serve as progenitors for adipocytes but also give rise to bone‐forming osteoblasts [3]. Adipogenesis within the bone marrow cavity is often associated with ageing and can accompany pathologies such as diabetes mellitus, impaired haematopoiesis or osteoporosis [4, 5, 6]. Thus, modulating adipogenesis in the bone marrow could serve as a potential therapeutic strategy for these conditions [7, 8].

Although BMAds exhibit distinct characteristics compared to those in peripheral white adipose tissue (WAT) [9, 10], the fundamental process by which both types differentiate from stem/progenitor cells into mature adipocytes is remarkably similar [11, 12]. This process, known as adipogenesis or adipogenic differentiation, involves a complex cascade of molecular events that ultimately lead to the development of lipid‐laden, mature adipocytes. Several murine cell culture models are available for studying in vitro adipogenic differentiation, which including primary BMSCs, the bone marrow stromal cell line ST2, the mesenchymal cell line C3H/10 T1/2 and the preadipocyte cell line 3 T3‐L1 [13, 14]. At early adipogenesis, adipogenic cocktail activates CCAAT/enhancer binding proteins (C/EBP) β and C/EBPδ, leading to the subsequent transcriptional activation of the peroxisome proliferator‐activated receptor (PPAR)γ and C/EBPα. This cascade progresses to the terminal stage of lipid accumulation and upregulates the lipogenic transcription factor (TF) sterol regulatory element‐binding protein 1 (Srebp1), which subsequently initiates lipogenesis [15].

Foxk1 and Foxk2 (collectively referred to as Foxks) are members of the Forkhead TF family, which are ubiquitously expressed across various tissues and organs and play pivotal roles in cellular regulation. Structurally, Foxks are highly conserved and possess a Forkhead box (Fox) domain that primarily recognises and binds to promoter regions containing the canonical sequence 5'‐GTAACA‐3′. Functionally, Foxks are implicated in a range of diseases and have complex roles in tumorigenesis, serving as critical transcriptional regulators [16]. Foxks recruit Sin3A‐HDAC complexes to inhibit acetylation of histone H4 and suppress the expression of key autophagy genes, thereby repressing starvation‐induced atrophy and autophagy programs [17]. Additionally, they facilitate the nuclear translocation of DVL, thereby activating Wnt/β‐catenin signalling, which promotes colorectal cancer development [18]. Furthermore, Foxks exhibit reciprocal regulation with Foxo1, modulating their localisation between the cytoplasm and nucleus in response to insulin signalling. This mutually exclusive localisation underscores their role in mediating the insulin signalling pathway [19]. Our study on adipocyte differentiation of BMSCs has demonstrated that Foxk1 facilitates this process by enhancing the transcriptional activity of the PPARγ2 promoter [20]. Moreover, Foxk1 has been shown to suppress hepatic fatty acid oxidation in an mTORC1‐dependent manner, thereby enhancing lipid catabolism in hepatocytes [21]. In addition, Sukonina et al. observed that overexpression of either Foxk1 or Foxk2 in 3 T3‐L1 pre‐adipocytes enhances glucose uptake and de novo triglyceride synthesis [22]. Despite these findings, the role of Foxk2 in adipogenic differentiation remains unexplored, warranting further investigation.

In the present study, we investigated the expression of Foxk2 and its novel role in primary BMSCs, as well as in the cell lines C3H/10 T1/2, ST2, and 3 T3‐L1 during adipogenic differentiation. We identified Foxk2 as a crucial transcriptional regulator of adipogenesis. Mechanistically, our findings demonstrate that Foxk2 translocates to the nucleus upon adipogenic stimulation and directly binds to the promoters of Pparγ1 and Pparγ2, thereby enhancing their transcriptional activity to promote adipocyte differentiation. Our results suggest that Foxk2 plays a key role in adipocyte differentiation in vitro, and that the Foxk2‐Pparγ axis may serve as a potential therapeutic target in the continuum of aging‐associated or oestrogen lose‐induced bone marrow adipose accumulation and osteoporosis.

## 2 Materials and Methods

### 2.1 Mice

8‐week‐old male C57BL/6J mice, db/db and db/m mice were purchased from HFK bioscience company (Beijing, China). The mice were performed via cervical dislocation following carbon dioxide inhalation. The murine tissues were collected and snap frozen in liquid nitrogen.

### 2.2 Cell Culture

3 T3‐L1, C3H/10 T1/2, and 293A cells were cultured in DMEM with 10% fetal bovine serum (FBS), while ST2 cells and primary bone marrow stromal cells (BMSCs) were maintained in α‐MEM with 10% FBS. BMSCs were isolated from the femurs and tibiae of 6‐week‐old C57BL/6J mice, as previously described [20]. Briefly, bone marrow cells were collected and cultured, and adherent cells were expanded until they reached 90% confluence. Cells from passages two to four were used for subsequent experiments. All cultures were maintained in a humidified incubator at 37°C with 5% CO₂.

For adipogenic differentiation, 90% confluent cells were cultured in adipocyte‐inducing medium (AIM, corresponding cell culture medium containing 10% FBS, 0.25 μM dexamethasone, 0.125 mM methylisobutylxanthine, 5 μg/mL insulin, and 50 μM indomethacin) for 72 h, followed by treatment for an additional 48 h with 5 μg/mL insulin alone.

### 2.3 Reagents

For the Western blot analysis, anti‐β‐actin, anti‐Histone H3 was purchased from ABclonal Technology (WuHan, China); anti‐Foxk1 (#12025), anti‐Foxk2 (#28712), anti‐C/EBPα (#2295), anti‐PPARγ (#2443), anti‐Foxo1 (#2880) and anti‐FABP4 (#2120) were purchased from Cell Signalling Technology (Danvers, MA, USA); and anti‐SREBP1 (14088‐1‐AP), anti‐FASN (10624‐2‐AP), and anti‐PERILIPIN (83905‐4‐RR) were purchased from Proteintech (Wuhan, China). Lipofectamine RNAiMAX was purchased from Invitrogen (Carlsbad, CA, USA), and Attractene Transfection Reagent was purchased from QIAGEN (Hilden, Germany). PI3‐kinase inhibitor (PI3K) LY294002, Akt inhibitor MK‐2206 dihydrochloride, and the mTOR inhibitor rapamycin were purchased from MedChemExpress (Shanghai, China).

### 2.4 Cell Transfection

siRNAs targeting the coding sequences of Foxk2 and PPARγ2 were designed and synthesised by GenePharma (Shanghai, China). Oligonucleotide and plasmid transfection was performed with Lipofectamine RNAiMAX or Attractene Transfection Reagent according to the manufacturer's protocol. Briefly, cells were seeded in plates the day before transfection to ensure suitable cell confluence on the day of transfection. For oligonucleotides, 50 nM siRNA or its negative control RNA (si‐NC) was used. For plasmid DNA, 0.5 μg DNA/well was used in a 24‐well plate or 1.0 μg DNA/well was used in a 12‐well plate.

### 2.5 5‐Ethynyl‐2′‐Deoxyuridine Proliferation Assay

For the proliferation assay, EdU staining was conducted using the BeyoClick EdU Cell Proliferation Kit with Alexa Fluor 594 or 488 (Beyotime, Shanghai, China) according to the manufacturer's protocol. In brief, C3H/10 T1/2, ST2, or BMSCs were incubated with 25 μM or 50 μM EdU for 2–4 h, followed by fixation with 4% paraformaldehyde for 15 min. Cells were then permeabilised with 0.3% Triton X‐100 for 10 min. Subsequently, they were incubated with Click Additive Solution for 30 min, and nuclei were counterstained with Hoechst 33342 for 10 min.

### 2.6 Oil Red O Staining

Adipocyte‐differentiated cells were gently washed twice with cold 1 × PBS and subsequently fixed with 4% paraformaldehyde for 10 min. The cells were then rinsed twice with 1 × PBS and stained with 60% saturated Oil Red O solution for 5 min. For quantification, the dye was extracted using isopropanol, and absorbance was measured at 520 nm after rocking the plate on a shaker for 15 min.

### 2.7 Quantitative PCR

Total RNA was extracted from cells or mouse tissues using a Total RNA Isolation Kit (Omega Bio‐Tek, Norcross, GA, USA). For each sample, 0.5 μg of RNA was reverse‐transcribed into cDNA using a Reverse Transcription Kit (Thermo Fisher, Shanghai, China). Quantitative PCR (qPCR) analysis was conducted using the 2X Universal SYBR Green Fast qPCR Mix (ABclonal Technology Co. Ltd., Wuhan, China) on a Light Cycler 96 Real‐Time PCR System (Roche Diagnostics Ltd., Mannheim, Germany). mRNA levels were normalised to Actb as internal controls, and relative expression levels were calculated using the comparative threshold cycle method (ΔΔ^C^_t_). Primer sequences used in this study are listed in Table 1.

**TABLE 1:** Sequences of oligonucleotides used in this article.

Name	Sequence
**Primer for RT‐qPCR analyses to mRNA**	
Foxk2‐F	5′– CCA TAC TAC AGG ACT GCG GAC A − 3′
Foxk2‐R	5′– CAG GGT CTA TCC TCC AGA AGG A − 3′
C/ebpα‐F	5′– CGG GAA CGC AAC AAC ATC G − 3′
C/ebpα‐R	5′– CGT GTC CAG TTC ACG GCT CA −3′
Pparγ‐F	5′– GGA AGC CCT TTG GTG ACT TTA TGG −3′
Pparγ‐R	5′– TGC AGC AGG TTG TCT TGG ATG T − 3′
Pparγ1‐F	5′– CAA GAT TTG AAA GAA GCG GTG A − 3′
Pparγ2‐F	5′– TGT TAT GGG TGA AAC TCT GGG A − 3′
Pparγ1&2‐R	5′– TGC TGG AGA AAT CAA CTG TGG T − 3′
Fabp4‐F	5′– AAA TCA CCG CAG ACG ACA GG −3′
Fabp4‐R	5′– GGC TCA TGC CCT TTC ATA AAC –3′
Actb‐F	5′– CTT CTT TGC AGC TCC TTC GTT G − 3′
Actb‐R	5′– CCT TCT GAC CCA TTC CCA CC –3′
Srebp1c‐F	5′– AAGCTGTCGGGGTAGCGTCT −3′
Srebp1c‐R	5′– CGGGAAGTCACTGTCTTGGTTGT −3′
Acly‐F	5′– AGGAAGTGCCACCTCCAACAGT −3′
Acly‐R	5′– CGCTCATCACAGATGCTGGTCA −3′
Fasn‐F	5′– CACAGTGCTCAAAGGACATGCC –3′
Fasn‐R	5′– CACCAGGTGTAGTGCCTTCCTC –3′
Scd1‐F	5′– GCAAGCTCTACACCTGCCTCTT −3′
Scd1‐R	5′– CGTGCCTTGTAAGTTCTGTGGC –3′
Fsp27‐F	5′– TCGGAAGGTTCGCAAAGGCATC –3′
Fsp27‐R	5′– CTCCACGATTGTGCCATCTTCC – 3′
Lpl‐F	5′– GCT GTA ACA ATC TGG GCT ATG − 3′
Lpl‐R	5′– TTG CTT GCC ATC CTC AGT C – 3′
**siRNA sequences**	
siFoxk1‐sence	5′– UCU AUU CGC CAA AAA GAG CCC – 3′
siFoxk1‐antisense‐globally	5′– GCU CUU UUU GGC GAA UAG ACC –3′
siFoxk2‐1‐sence	5′– GCC ACA AUC UCU CUC UGA ATT −3′
siFoxk2‐1‐antisense‐globally	5′– UUC AGA GAG AGA UUG UGG CTT −3′
siFoxk2‐2‐sence	5′– UUA CAG UUU UUA UUG GAA GCU –3′
siFoxk2‐2‐antisense‐globally	5′– CUU CCA AUA AAA ACU GUA ACG −3′
siPparγ‐sence	5′– UUU AUU UCU ACU UUU UUU GUG −3′
siPparγ‐antisense‐globally	5′– CAA AAA AAG UAG AAA UAA AUG −3′
**Primers for ChIP qPCR assay**	
Pparγ1 promoter—site #I‐F	5′– AGG TCT CCT ATG TAA GAA ATG GTG CTA A − 3′
Pparγ1 promoter—site #I‐R	5′– TGG TGC TGG GAA CCG AAC A − 3′
Pparγ1 promoter—site #II‐F	5′– GCA TTC GCC TTC ATA ACA TTC TG − 3′
Pparγ1 promoter—site #II‐R	5′– AAT TAA GTG AAT TTA GGA TCT CCC TTT T − 3′
Pparγ1 promoter—site #III‐F	5′– CAT ATA AAT ATA TAT ATG GGT GTG TAT CT − 3′
Pparγ1 promoter—site #III‐R	5′– GCT TTT CAT CTC TCT CTC TAT ATA TAT ACC – 3′
Pparγ2 promoter—site #I‐F	5′– CTG GTA ATA CAT TAT TCT CAG GGA − 3′
Pparγ2 promoter—site #I‐R	5′– TTG TTT ATC TAT CAA TAG CAA CTG C – 3′
Pparγ2 promoter—site #II‐F	5′– GTC AGG ACA GTG CCA GCC AAT − 3′
Pparγ2 promoter—site #II‐R	5′– GGC TTA TGG TCA TCG AGC TTA TGA − 3′

Abbreviations: F, forward; R, reverse.

### 2.8 Construction of Plasmids, and Adenovirus Packaging and Transduction

For overexpression studies, the sequences of Foxk2 were synthesised by Sangon Biotech (Shanghai, China) and subcloned into either the pCDNA3.1(+) vector at the AflII/EcoRV sites or the pENTR 3C vector (Invitrogen, Carlsbad, CA, USA) at the SalI/EcoRV sites. An adenoviral expression construct for Foxk2 was then generated by inserting the Foxk2 sequence into the adenoviral vector Ad/CMV/V5‐DEST using Gateway technology (Invitrogen, Carlsbad, CA, USA). The control adenoviral expression construct was generated by inserting the eGFP sequence into the Ad/CMV/V5‐DEST vector. To construct the C/EBPα, PPARγ1, and PPARγ2 expression vectors, the coding sequences of these genes were first amplified by PCR and then recombined with their respective vectors using MultiF Seamless Assembly Mix (ABclonal Technology).

Adenoviruses were generated in 293A cells following the manufacturer's protocol (ViraPower Adenoviral Expression System, Invitrogen) and used at an MOI of 200 to infect C3H/10 T1/2 cells.

The PPARγ1 and PPARγ2 promoter‐containing vectors were constructed as described previously [20]. For the construction of the Foxk2 luciferase reporter plasmid, a wild‐type mouse Foxk2 promoter fragment (−1826 to +29) was amplified by PCR and cloned into the respective vector at the KpnI/HindIII sites.

The sequences of primers and complementary oligonucleotides used in this study are provided in Table 1.

### 2.9 Immunofluorescence Staining

C3H/10 T1/2 cells were infected with Ad‐GFP or Ad‐Foxk2 for 15 h, then cultured in basal medium or AIM for 48 h. Following this, cells were washed and fixed with 4% paraformaldehyde for 15 min, permeabilised with 0.2% Triton X‐100 for 15 min, and blocked with 5% bovine serum albumin for 30 min. The cells were then incubated overnight at 4°C with a Foxk2 antibody, washed three times with 1× PBS, and incubated with Alexa Fluor 488 or 594‐conjugated secondary antibody (ZSGB‐BIO, Beijing, China) for 2 h. Afterwards, nuclei were stained with DAPI (Sigma‐Aldrich) for 5 min. Fluorescence signals were captured using confocal laser scanning microscopy (ZEISS LSM 800; Germany).

### 2.10 Cell Nucleus/Cytoplasm Fractionation

For cytoplasmic fraction preparation, cells were washed twice with cold 1× PBS and then lysed in 1% NP40 1× PBS containing a protease inhibitor cocktail (Sigma–Aldrich, St. Louis, MO, USA). Following a brief centrifugation, the supernatant was collected as the cytoplasmic fraction. The pellet, after additional washing, was considered the nuclear fraction.

### 2.11 Western Blotting

Treated cells were lysed using Cell lysis buffer for Western and IP (Beyotime, Shanghai, China), and immunoblotting was performed as previously described [20]. Briefly, proteins were separated by 10% SDS‐PAGE and transferred onto a polyvinylidene difluoride membrane. The membranes were blocked with 5% skim milk and incubated with the appropriate primary antibodies at 4°C overnight. Following washing, the membranes were incubated with horseradish peroxidase (HRP)‐conjugated secondary antibodies (1: 3000; Simu Biotech, Tianjin, China). The protein of interest was visualised using Immobilon Western Chemiluminescent HRP Substrate (Millipore, Billerica, MA, USA).

### 2.12 Dual‐Luciferase Reporter Assay

C3H/10 T1/2 cells were seeded in a 96‐well plate at 5000 cells per well the day before transfection. The cells were transfected with firefly luciferase reporter vectors, the Renilla luciferase control vector pRL‐SV40 (Promega, Madison, USA), and either C/EBPβ, C/EBPδ, Klf5, C/EBPα, PPARγ1 or PPARγ2 expression plasmids. After 48 h, luciferase activity was measured using a dual‐luciferase reporter assay kit (Promega, Madison, USA).

### 2.13 Chromatin Immunoprecipitation Assay

C3H/10 T1/2 cells were infected with Ad‐GFP or Ad‐Foxk2 for 48 h, then harvested for chromatin immunoprecipitation (ChIP) assays using a Sonication ChIP Kit (ABclonal Technology Co. Ltd., Wuhan, China). Chromatin was digested with micrococcal nuclease and then sheared by sonication. Soluble chromatin was incubated with 2.5 μg of rabbit anti‐Foxk2 antibody or rabbit IgG, followed by binding to protein A/G magnetic beads. The DNA was decross‐linked and subsequently used for qPCR to amplify mouse PPARγ1 and PPARγ2 promoter‐specific sequences. Primer sequences are listed in Table 1.

### 2.14 Statistical Analysis

Results are presented as mean ± standard deviation (S.D.). Differences between two groups were analysed using a two‐tailed Student's *t*‐test, while differences among multiple groups were assessed using one‐way ANOVA followed by Tukey's post hoc test. Statistical analysis was conducted with SPSS software (version 26). Significance was defined as *p* < 0.05, with statistical significance indicated by asterisks: *p < 0.05, **p < 0.01, ***p < 0.001 and ****p < 0.0001.

## 3 Results

### 3.1 Foxk2 Was Enhanced in iWAT and eWAT of Obese Mice

**FIGURE 1:**
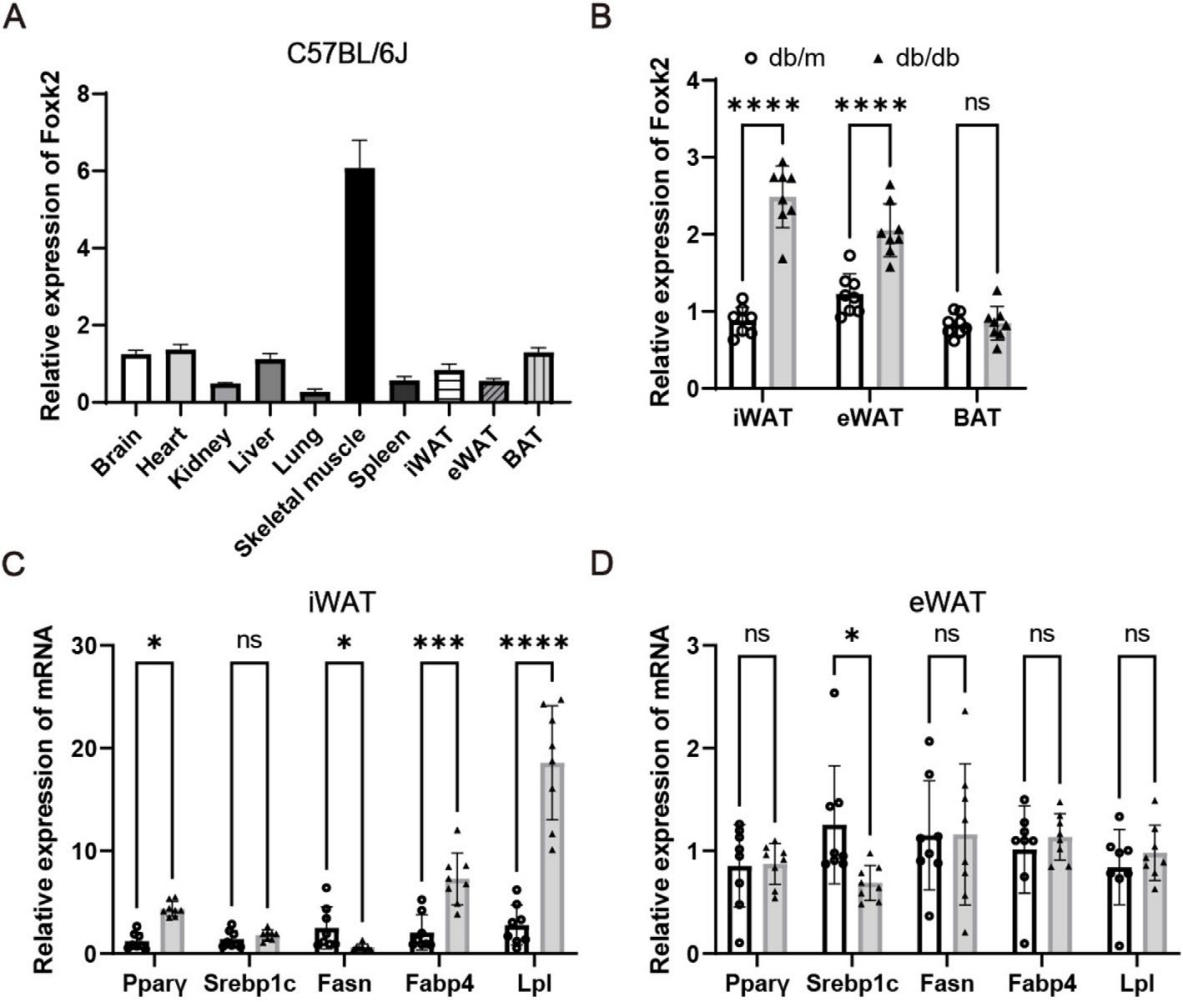
Foxk2 was enhanced in iWAT and eWAT of obese mice. (A) RT‐qPCR analysis was conducted to quantify Foxk2 expression across various tissues in C57BL/6J mice (*n* = 4). The expression level of Foxk2 in the liver was normalised to 1. (B‐D) RT‐qPCR was used to assess the relative expression of Foxk2, PPARγ, Srebp1c, Fasn, Fabp4, and Lpl in specific adipose tissues. Data are presented as mean ± S.D. (*n* = 8). Statistical significance was determined using a two‐tailed Student's *t*‐test with significance levels indicated as *p* < 0.05 (*), p < 0.001 (***), and *p* < 0.0001 (****). Values are presented relative to non‐obese db/m mice.

We detected the expression profile of Foxk2 in various tissues of 8‐week‐old C57BL/6J mice. As shown in Figure 1A, Foxk2 mRNA level was most abundant in skeletal muscle, moderate in brain, heart, liver, spleen, inguinal (iWAT), epididymal white adipose tissue (eWAT) and interscapular brown adipose tissue (BAT), and relatively low level in kidney and lung. Moreover, we found that Foxk2 expression levels were induced by obesity in mice iWAT and eWAT (Figure 1B, obese db/db mice vs. lean db/m mice). In addition, adipocyte markers Pparγ, Fabp4 and Lpl were higher in iWAT but not in eWAT of obese db/db mice compared to non‐obese db/m mice (Figure 1C,D). These results indicate that upregulated Foxk2 expression is positively related to adipose expansion or accumulation.

### 3.2 Adipogenic Induction Stimulates Foxk2 Expression in Progenitor Cells

**FIGURE 2:**
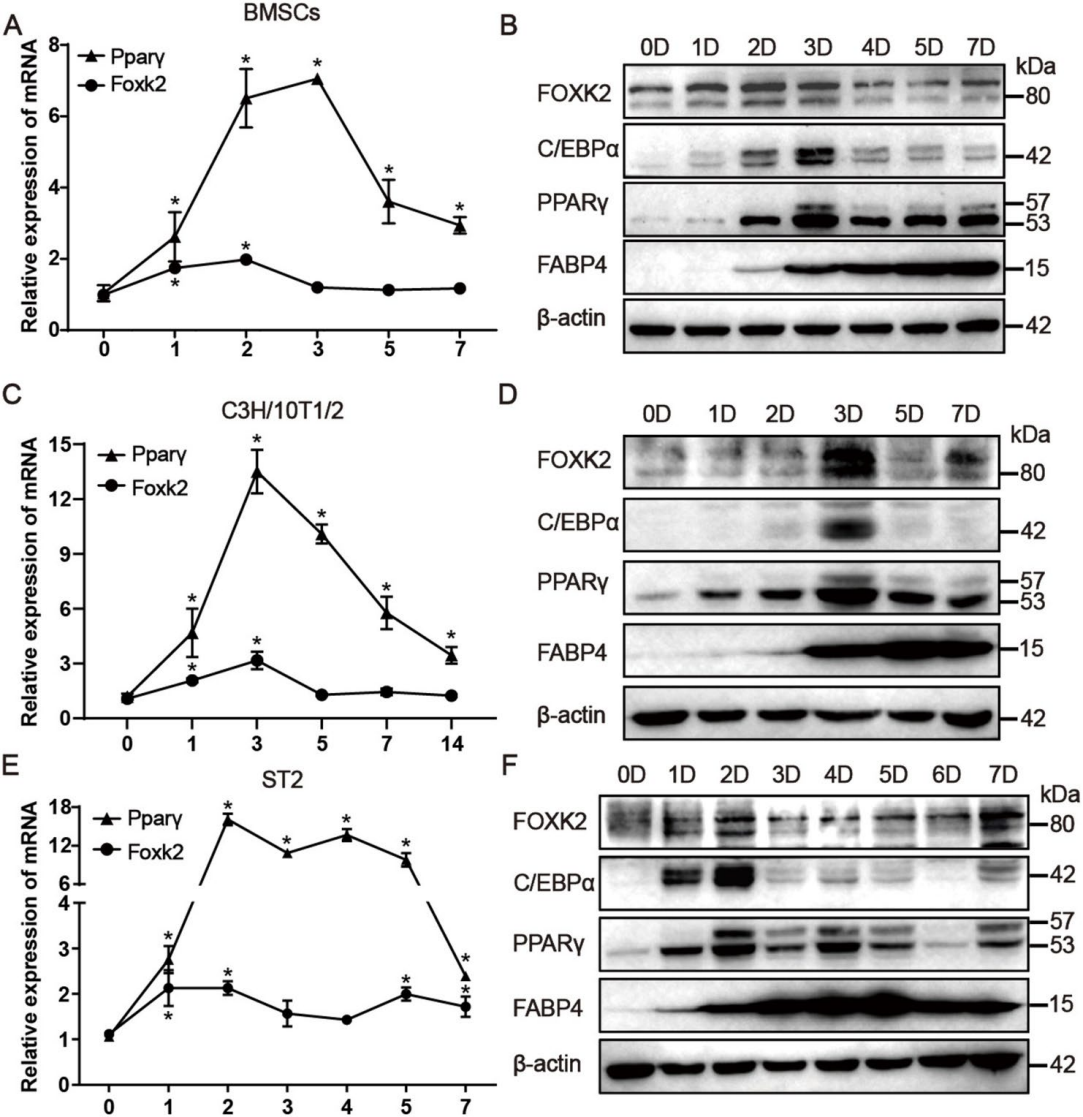
Adipogenic induction stimulates Foxk2 expression in progenitor cells. (A, C, E) RT‐qPCR was used to measure the mRNA expression levels of PPARγ and Foxk2 in primary BMSCs (A), C3H/10 T1/2 cells (C), and ST2 cells (E) at specified time points during adipogenic differentiation. (B, D, F) Western blotting analysis was performed to assess the protein levels of Foxk2, C/EBPα, PPARγ, and FABP4 in primary BMSCs (B), C3H/10 T1/2 cells (D), and ST2 cells (F) at the same time points. Data are presented as means ± S.D. Statistical significance was determined using ANOVA followed by Tukey's test, with significance indicated as *p < 0.05, compared to the control group.

To delineate the role of Foxk2 in adipogenic differentiation, we first analysed its expression pattern in primary BMSCs, progenitor cell line C3H/10 T1/2 cells and ST2 cells during their commitment to the adipocyte lineage. In differentiated BMSCs, C3H/10 T1/2 and ST2 cells, an increasing trend in Pparγ expression was observed compared to day 0, with peaks occurring on days 3, 3, and 2, respectively (Figure 2A,C,E). Similarly, the expression of Foxk2 was also enhanced during adipogenic differentiation, peaking on day 2, 3, and 2. The peak expression of Foxk2 either preceded or coincided with that of Pparγ. Furthermore, FOXK2 and adipocyte marker proteins (C/EBPα, PPARγ and FABP4) were upregulated following adipogenic induction (Figure 2B,D,F). Correspondingly, the protein expression patterns of FOXK2 and PPARγ also showed more similar trends.

### 3.3 Foxk2 Promotes Adipocyte Differentiation and Lipogenesis of Progenitor Cell Lines

**FIGURE 3:**
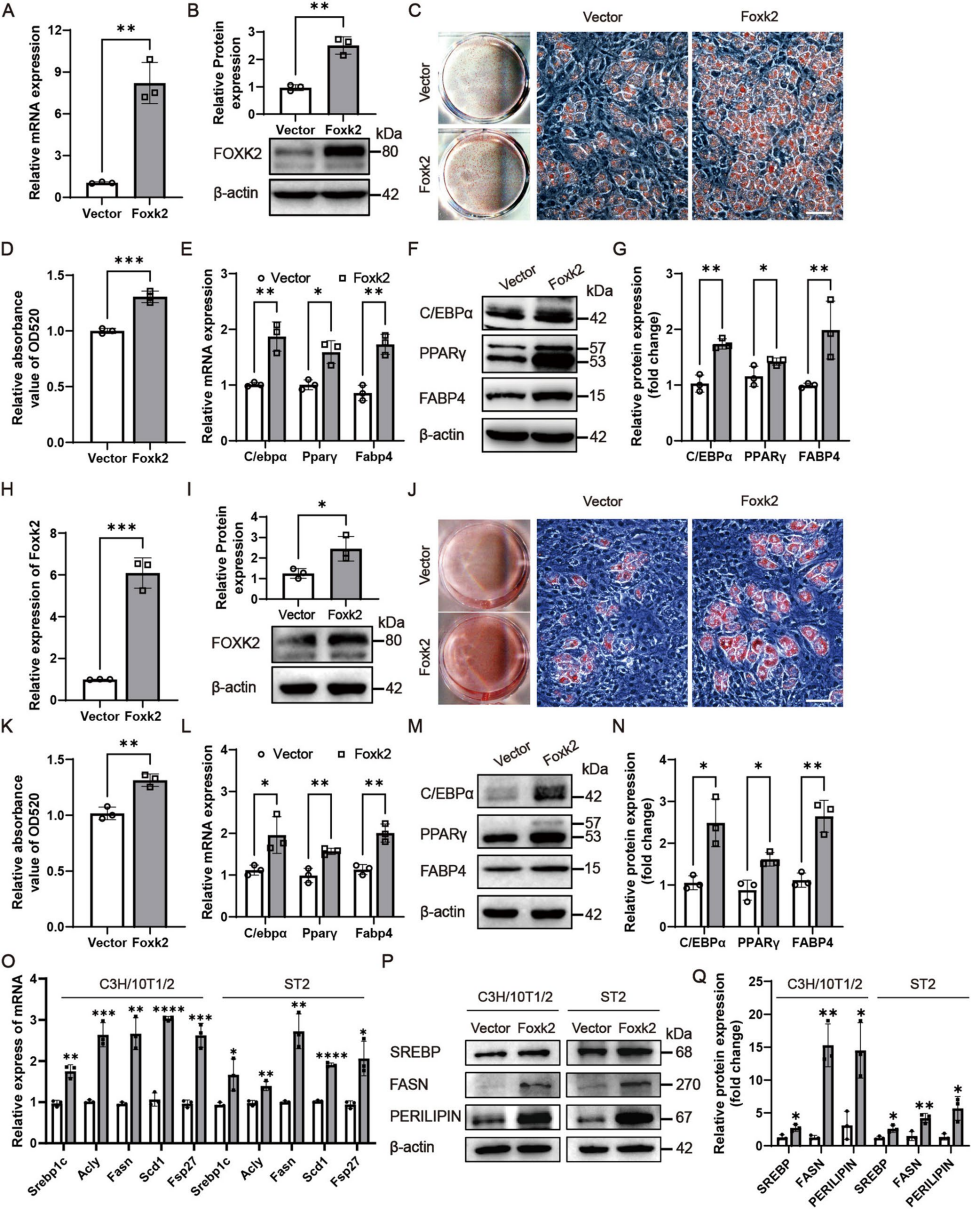
Foxk2 promotes adipocyte differentiation and lipogenesis of progenitor cell lines. (A, B, H, I) RT‐qPCR and Western blotting were conducted to evaluate Foxk2 expression in C3H/10 T1/2 (A, B) and ST2 cells (H, I) 2 days after transfection with Foxk2 coding constructs. (C–G, J–Q) Transfected C3H/10 T1/2 (C–G, O–Q) and ST2 cells (J–Q) were induced in AIM for 5 days, followed by Oil Red O staining, and representative images of the stained cells are shown (C, J). The stain extracted with isopropanol was measured at 520 nm by spectrophotometry (D, K). Next, RT‐qPCR (E, L, O) and Western blotting (F, M, P) were performed to assess the expression of adipocyte differentiation genes C/EBPα, PPARγ, and FABP4, along with lipogenesis‐specific genes Srebp1c, Acly, Fasn, Scd1, Fsp27, and Perilipin. Western blot bands were quantified by greyscale analysis (G, N, Q). All images were captured using a light microscope with a scale bar of 100 μm (C, J). β‐actin was used as an internal control. Data are presented as mean ± S.D. Statistical significance was determined using two‐tailed Student's *t*‐test, with significance indicated as *p < 0.05, **p < 0.01, ***p < 0.001, and ****p < 0.0001 compared to the control group.

To clarify the role of Foxk2 in adipocyte differentiation, we first transfected its expression construct into C3H/10 T1/2 and ST2 cells, successfully upregulating both the mRNA and protein levels of Foxk2, as confirmed by qRT‐PCR (Figure 3A,H) and western blotting (Figure 3B,I). Overexpression of Foxk2 enhanced the proliferation (Figure S1A,C,) and adipogenic differentiation of C3H/10 T1/2 and ST2 cells, as evidenced by increased Oil Red O staining (Figure 3C,D for C3H/10 T1/2, and Figure 3J,K for ST2 cells). Additionally, adipogenic treatment significantly upregulated the mRNA and protein levels of C/ebpα, Pparγ, and Fabp4 in Foxk2‐overexpressing cells (Figure 3E–G for C3H/10 T1/2, and Figure 3L–N for ST2 cells). Foxk2 also affected expression levels of lipogenic factors in C3H/10 T1/2 and ST2 cells. Enhanced expression of Foxk2 significantly increased the mRNA levels of lipogenic genes (Srebp1c, Acly, Fasn, and Scd1) and the lipid droplet‐containing protein Fsp27 (Figure 3O). Consistently, protein levels of SREBP, FASN, and PERILIPIN were also upregulated (Figure 3P,Q). Moreover, while overexpression of either Foxk1 or Foxk2 significantly promoted the differentiation of C3H/10 T1/2 and ST2 cells into adipocytes, co‐overexpression of both did not result in any further enhancement of this effect (Figure S2A,B).

### 3.4 Inhibition of Foxk2 Hindered Adipocyte Differentiation and Lipogenesis in Progenitor Cell Lines

**FIGURE 4:**
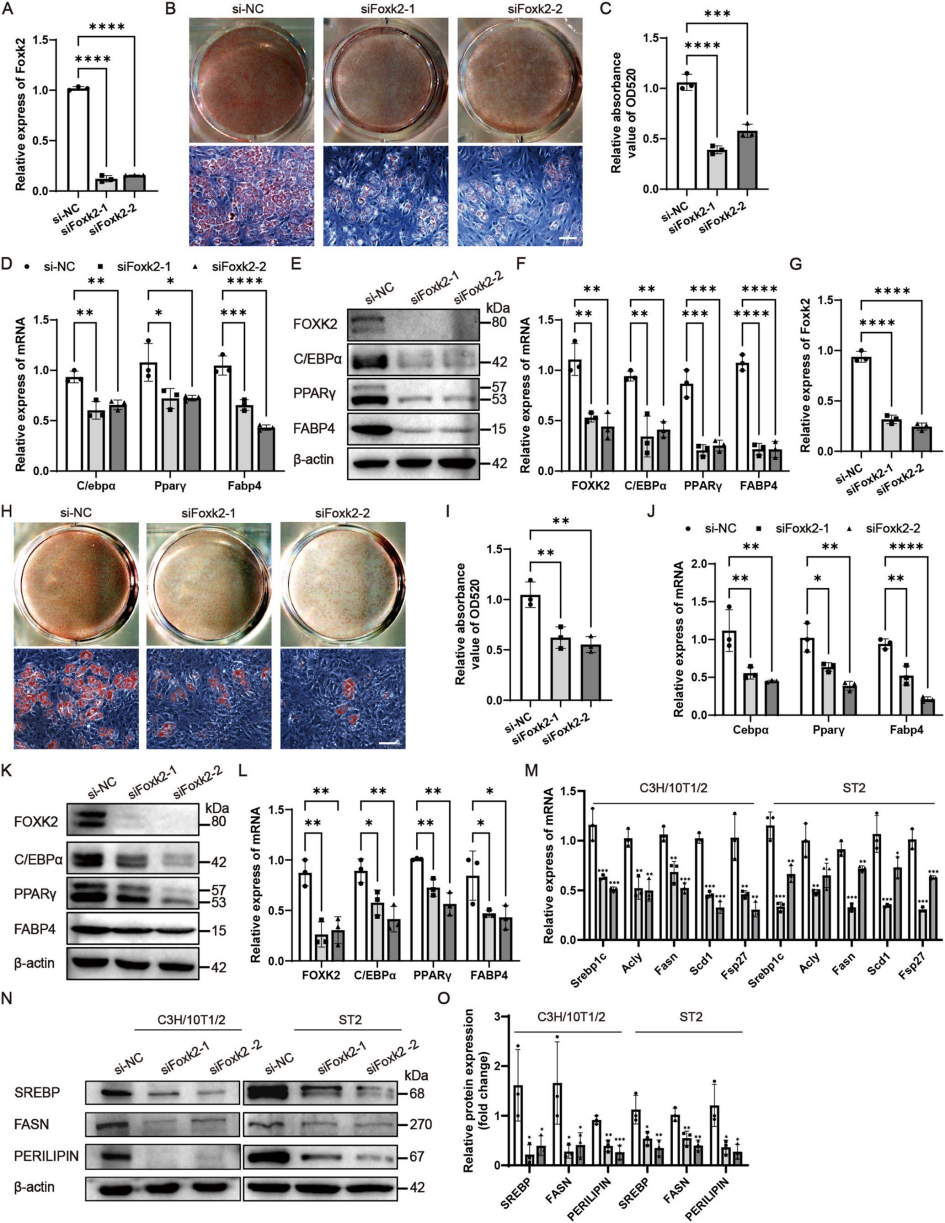
Inhibition of Foxk2 hindered adipocyte differentiation and lipogenesis in progenitor cell lines. A–O, the effects of Foxk2 silencing on adipocyte differentiation and lipogenesis of C3H/10 T1/2 (A–F, M–O) and ST2 (G–O) cells were studied. Foxk2 Silencing after transfection of Foxk2 siRNAs (siFoxk2‐1, siFoxk2‐2) or negative control RNA (si‐NC) was verified using RT‐qPCR (A, G). Oil Red O staining was performed in differentiated adipocytes (B, H) and the stain extracted with isopropanol was measured at 520 nm by spectrophotometry (C, I). The mRNA (D, J, M) and protein (E, K, N) levels of adipogenic factors were measured 3 days after adipogenic treatment. Western blot bands were quantified by greyscale analysis (F, L, O). All images were captured using a light microscope with a scale bar of 100 μm in (B, H). β‐actin served as an internal control. Data are presented as mean ± S.D. Statistical significance was determined using a two‐tailed Student's *t*‐test, with significance indicated as *p < 0.05, **p < 0.01, ***p < 0.001, and ****p < 0.0001 compared to the control group.

To verify the role of Foxk2 in adipogenic differentiation and lipogenesis, we conducted a loss‐of‐function study using small interfering RNAs (siRNAs). C3H/10 T1/2 and ST2 cells were transfected with either of two distinct Foxk2 siRNAs or a negative control siRNA (si‐NC) for 48 h. As expected, transfection with the two siRNAs substantially reduced endogenous Foxk2 expression (Figure 4A,G). The reduction in Foxk2 led to decreased proliferation of C3H/10 T1/2 and ST2 cells (Figure S1B,D). Additionally, Foxk2 knockdown significantly suppressed adipocyte formation following adipogenic treatment, as evidenced by attenuated Oil Red O staining (Figure 4B,C,H,I). Further validation by RT‐qPCR and immunoblotting confirmed lower expression levels of C/ebpα, Pparγ, and Fabp4 (Figure 4D–F,J–L). We also examined the effect of Foxk2 knockdown on the expression levels of lipogenic genes in C3H/10 T1/2 and ST2 cells. As expected, the mRNA levels of lipogenic genes Srebp1c, Acly, Fasn, Scd1, and lipid droplet‐containing protein Fsp27 were decreased by downregulated expression of Foxk2 72 h after adipogenic treatment (Figure 4M). Consistently, the protein levels of SREBP, FASN, and PERILIPIN were also reduced (Figure 4N,O). Furthermore, to explore the synergistic effects of Foxk1 and Foxk2 in adipocyte differentiation and lipogenesis, Foxk1 and Foxk2 siRNAs were transfected into C3H/10 T1/2 cells, either individually or simultaneously. We found that transfection of either Foxk1 or Foxk2 siRNAs significantly inhibited the differentiation of C3H/10 T1/2 cells into adipocytes; however, co‐transfection of both siRNAs did not further enhance this inhibition (Figure S2C–F). Above findings reveal that downregulation of Foxk2 inhibits adipogenic differentiation and lipogenesis, and Foxk1 and Foxk2 may exhibit functional redundancy in this process.

### 3.5 Reduced Expression of Foxk2 Impaired Adipocyte Differentiation and Lipogenesis in 3 T3‐L1 Preadipocytes

Next, under adipogenic condition, knockdown of Foxk2 also significantly suppressed adipocyte formation from 3 T3‐L1 preadipocytes (Figure S3A). Oil Red O quantification revealed 61% and 55% decrease, respectively, after siRNA transfection compared to si‐NC (Figure S3B). Correspondingly, reduced Foxk2 expression led to decreased mRNA levels of adipogenic TFs and marker genes, including Pparγ, C/ebpα, and Fabp4, as well as lipogenic genes (Srebp1c, Acly, Fasn, and Scd1) (Figure S3C,D). This reduction was also reflected at the protein level (Figure S3E,F).

### 3.6 Foxk2 Induced Adipogenic Differentiation in Primary BMSCs

**FIGURE 5:**
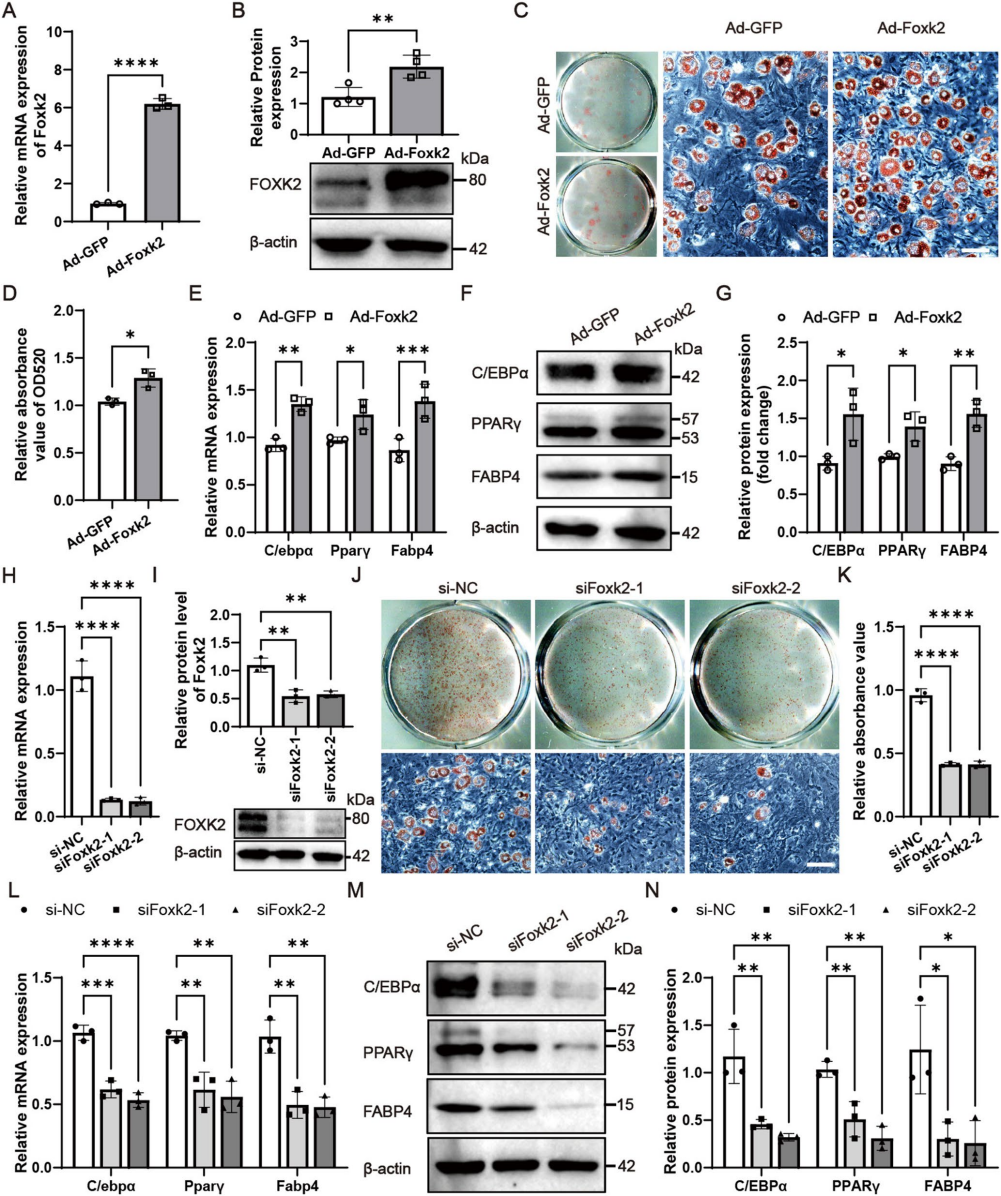
Foxk2 induced adipogenic differentiation in primary BMSCs. The overexpression (A, B) or knockdown (H, I) of Foxk2 was verified using RT‐qPCR and Western blotting in primary BMSCs after infection of Ad‐Foxk2 or transfection of Foxk2 siRNAs. The infected (C–G) or transfection (J–N) BMSCs were then cultured in AIM, and stained with Oil Red O after 5 days (C, J, scale bars represent 100 μm). The extent of Oil Red O staining was quantified by measuring absorbance at OD520 after extraction with isopropanol (D, K). After 3 days, RNA (E, L) and protein (F, M) levels of the adipocyte‐specific genes were assessed by RT‐qPCR and western blot analysis, respectively. Western blot bands were further quantified with greyscale analysis (G, N). β‐actin was used as an internal control. Data are presented as mean ± S.D., with statistical significance determined by a two‐tailed Student's *t*‐test: *p < 0.05, **p < 0.01, ***p < 0.001, and ****p < 0.0001 compared to the control group.

We further certify the positive effect of Foxk2 on adipogenesis in primary cultured mouse BMSCs. RT‐qPCR and Western blotting analyses revealed 6.6‐fold and 1.8‐fold increase in mRNA and protein levels, respectively, following infection with an adenovirus expressing Foxk2 (Figure 5A,B). Moreover, Foxk2 overexpression promoted the proliferation of BMSCs (Figure S1E). And, Foxk2 overexpression significantly enhanced adipogenesis following adipogenic treatment, as evidenced by a 24% increase in Oil Red O staining compared to control Ad‐GFP infection. (Figure 5C,D). Accordingly, the mRNA and protein levels of C/ebpα, Pparγ and Fabp4 were significantly elevated at 48 h and 72 h after adipogenic treatment in cells overexpressing Foxk2 (Figure 5E–G).

However, silencing of endogenous Foxk2 with siRNA in primary BMSCs reduced both cell proliferation (Figure S1F) and adipogenesis. After transfection for 2 days, Foxk2 knockdown efficiency was confirmed by RT‐qPCR and Western blotting (Figure 5H,I). After adipogenic treatment, Foxk2 siRNAs suppressed adipocyte formation (Figure 5J,K) and decreased the mRNA and protein levels of C/ebpα, Pparγ and Fabp4 compared to the negative control siRNA (Figure 5L–N). These results collectively demonstrate that Foxk2 plays a facilitative role in adipogenic differentiation.

### 3.7 Adipogenic Treatment Facilitated Foxk2 Nuclear Translocation

**FIGURE 6:**
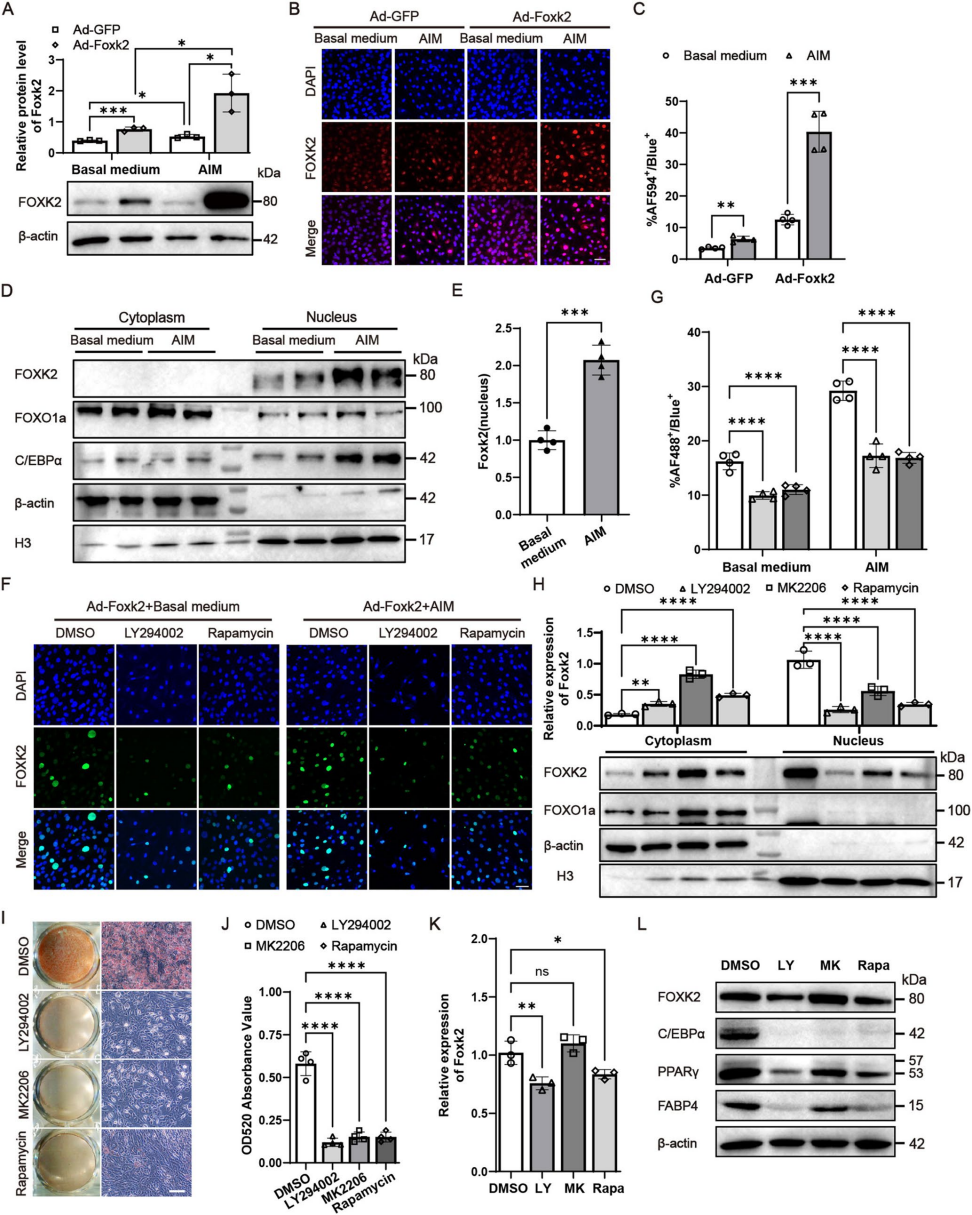
Adipogenic treatment facilitated Foxk2 nuclear translocation. (A) Western blotting was conducted to evaluate Foxk2 expression in C3H/10 T1/2 cultured by basal medium or AIM after 3 days. (B) The above cells immunostained for Foxk2 (AF594, red) and DAPI (blue). DAPI was used to label the nuclei. Scale bars represent 50 μm. (C) Frequency of Foxk2^+^ (AF594^+^) cells among DAPI^+^ cells. (D) Nuclear and cytoplasmic fractions were extracted from the Ad‐Foxk2‐infected cells, and the target genes in each fraction were assessed by immunoblotting. β‐actin and histone H3 were used as markers for the cytosolic and nuclear fractions, respectively. (E) Quantification of Foxk2 expression in the nuclear fraction was performed using greyscale analysis. (F) C3H/10 T1/2 cells infected with Ad‐Foxk2 and cultured in basal medium or AIM for 3 days in the presence or absence of LY294002 (10 μM), MK‐2206 (5 μM) or rapamycin (100 nM). Scale bars represent 50 μm. (G) Frequency of Foxk2^+^ (AF488^+^) cells among DAPI^+^ cells. (H) The target genes in nuclear and cytoplasmic fractions were assessed by immunoblotting. (I) The cells in (H) were also stained with Oil Red O, and representative images are shown. Scale bars represent 100 μm. (J) Oil Red O extracted with isopropanol was measured at OD520. (K, L) Protein levels of Foxk2 and the adipocyte‐specific genes were assessed by western blot analysis. The data are presented as mean ± S.D. *p < 0.05, **p < 0.01, ***p < 0.001, ****p < 0.0001 relative to the control group. A two‐tailed Student's *t*‐test was applied for comparisons between two groups, and one‐way ANOVA followed by Tukey's test was applied for comparisons among more than two groups.

We have found that adipogenic stimulation induces the nuclear localisation of Foxk1 [20], and our results indicate that Foxk2 functions similarly to Foxk1 in adipocyte differentiation. Thus, we asked whether Foxk2 functions in the same manner. Initially, C3H/10 T1/2 cells were infected with either control or Foxk2‐expressing adenovirus (Ad‐Foxk2), followed by treatment with basal or adipogenic medium. As shown in Figure 6A, Ad‐Foxk2 resulted in a marked increase in protein expression levels compared to control adenovirus, an effect further amplified by adipogenic induction. Additionally, cell immunofluorescence experiments revealed that Foxk2 exhibited robust translocation from the cytoplasm to the nucleus in response to adipogenic treatment, particularly when its expression was upregulated (Figure 6B,C). Next, cells infected with Ad‐Foxk2 and subsequently cultured in basal or adipogenic‐inducing medium were lysed and fractionated into cytoplasmic (marked by β‐actin) and nuclear (marked by histone H3) fractions. Foxk2 levels in each fraction were then analysed by immunoblotting (Figure 6D). Consistently, Foxk2 exhibited strong translocation from the cytoplasm to the nucleus (Figure 6D,E). Furthermore, in agreement with our previous report [20] significantly increased the nuclear levels of C/EBPα and PPARγ, while promoting the translocation of FoxO1 from the nucleus to the cytoplasm (Figure 6D).

We then used the PI3K inhibitor LY294002 (LY), Akt inhibitor MK‐2206 dihydrochloride (MK), and mTOR inhibitor rapamycin (Rapa) to block Foxk2 nuclear localisation following adipogenic treatment, as demonstrated by immunofluorescence staining (Figure 6F,G) and immunoblotting (Figure 6H). Consequently, Foxk2 retention in the cytoplasm impaired adipocyte differentiation (Figure 6I–L). These findings suggest that Foxk2 may regulate adipogenesis via nuclear translocation, likely mediated by the PI3K and mTOR signalling pathways.

### 3.8 Foxk2 Promoted Adipocyte Differentiation and Lipogenesis by Activating PPARγ Promoter

**FIGURE 7:**
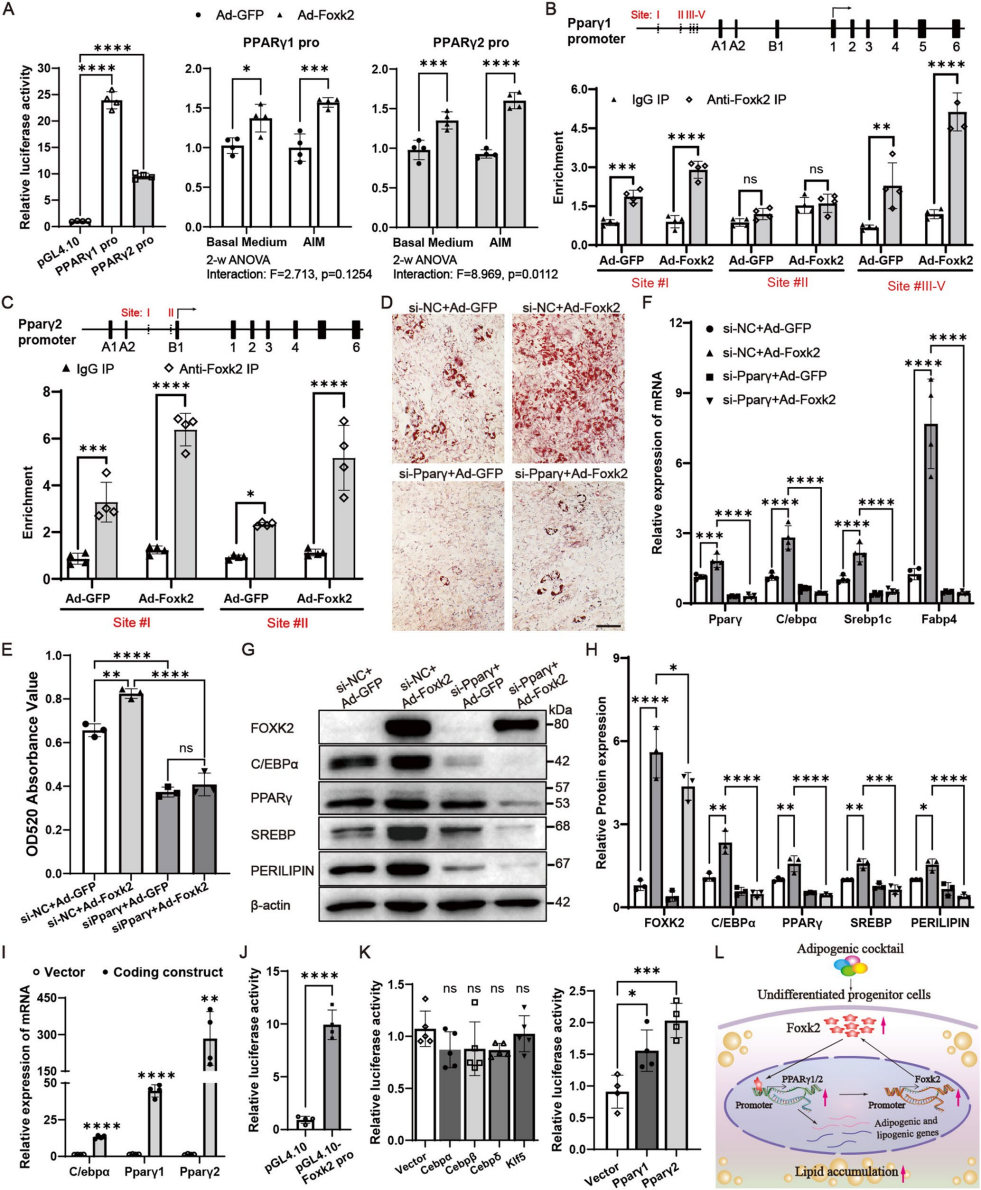
Foxk2 promoted adipocyte differentiation and lipogenesis by activating PPARγ promoter. (A) The activity of PPARγ1 and PPARγ2 promoter was verified in luciferase reporter constructions, and the transcriptional activity of PPARγ1 and PPARγ2 promoter in C3H/10 T1/2 cotransfected with Foxk2 expression construct was assayed. (B, C) ChIP assays were performed in C3H/10 T1/2 cells infected with adenovirus expressing Foxk2 to assess Foxk2 occupancy of the PPARγ1 (B) and PPARγ2 (C) promoters. (D–H) C3H/10 T1/2 cells were infected with Ad‐GFP or Ad‐Foxk2 and transfected with the indicated RNA duplex the following day. The cells were then cultured in AIM for 5 days, and stained with Oil Red O (D). Scale bars represent 100 μm. Oil Red O extracted with isopropanol was measured at OD520 (E). RT‐qPCR (F) and western blot analysis (G, H) were used to measure mRNA and protein levels of adipogenic genes. (I) RT‐qPCR was used to test the expression of C/ebpα, Pparγ1 or Pparγ2 in C3H/10 T1/2 cells 2 days after transfection with indicated coding construct. (J) Dual‐luciferase reporter assay was performed to assess the availability of the luciferase reporter vector containing the Foxk2 promoter in C3H/10 T1/2 cells. (K) Dual‐luciferase reporter assays were performed to test the effect of C/EBPα, C/EBPβ, C/EBPδ, KLF5, Pparγ1 or Pparγ2 overexpression on transcriptional activity of Foxk2 promoter in C3H/10 T1/2 cells. (L) Schematic model depicting Foxk2‐induced adipogenesis via PPARγ in vitro. The values are presented as means ± S.D. *p < 0.05, **p < 0.01, ***p < 0.001, ****p < 0.0001 relative to the control group. A two‐tailed Student's *t*‐test was applied for comparisons between two groups, and one‐way or two‐way ANOVA followed by Tukey's test was applied for comparisons or interaction among more than two groups.

We proceeded to investigate the mechanism by which Foxk2 enhances adipogenesis. Considering that PPARγ is a key regulator of adipocyte differentiation, and that Foxk1 has been shown to promote adipogenesis by partially activating the PPARγ2 gene transcription [20], we explored whether Foxk2 functions through a similar mechanism. Initially, our luciferase reporter gene assay revealed that, unlike Foxk1, which specifically activated only the pGL4.10‐PPARγ2 promoter, Foxk2 overexpression activated both the pGL4.10‐PPARγ1 and pGL4.10‐PPARγ2 promoters; additionally, Foxk2 overexpression led to a more pronounced enhancement of Pparγ2 promoter activity compared to Pparγ1 after adipogenic induction (Figure 7A). Next, ChIP followed by real‐time PCR confirmed Foxk2 binding to both the Pparγ1 and Pparγ2 promoters (Figure 7B,C). Foxk2 showed significant occupancy at the Pparγ1 promoter region at position −636 and − 321/265/241, as well as the Pparγ2 promoter region at position −756/712 and − 122. To determine whether Pparγ is involved in Foxk2‐mediated regulation of adipogenesis, we conducted gain‐of‐function experiments for Foxk2 in the context of Pparγ knockdown. C3H/10 T1/2 cells were first infected with Ad‐GFP or Ad‐Foxk2 and then transfected with Pparγ‐targeting siRNA. Following adipogenic treatment, the adipogenesis‐promoting effect of Foxk2 was significantly attenuated upon Pparγ reduction (Figure 7D,E). Consistently, the mRNA and protein levels of adipocyte differentiation and lipogenesis marker genes were lower in cells co‐treated with Ad‐Foxk2 and Pparγ siRNA compared to those treated with adenovirus alone (Figure 7F–H).

We also explored the role of key adipogenic TFs in regulating Foxk2 transcriptional activity, focusing on those induced during early adipogenesis, including C/EBPβ, C/EBPδ, Klf5, C/EBPα, Pparγ1 and Pparγ2. The overexpression efficiency of C/EBPβ, C/EBPδ and Klf5 was confirmed in our previous study [20]. In this study, the overexpression of C/EBPα, Pparγ1 and Pparγ2 (Figure 7I), along with the transcriptional activity of the pGL4.10‐Foxk2 promoter (Figure 7J), was validated using dual‐luciferase reporter assays and RT‐qPCR. Notably, only Pparγ1 and Pparγ2 significantly enhanced the transcriptional activity of the Foxk2 promoter (Figure 7K). This suggests that Foxk2 may promote adipogenesis via a Foxk2‐Pparγ positive feedback loop (Figure 7L).

## 4 Discussion

Accumulating evidence strongly supports that an overwhelming production of BMAT is a major contributor to bone loss disorders like osteoporosis [23, 24]. Therefore, BMAT‐targeted therapy can be an efficient and feasible intervention for osteoporosis. However, compared to blocking bone‐destroying molecules produced by BMAT, suppressing the formation of BMAT is theoretically a more effective and fundamental approach to treating osteoporotic bone diseases [7, 25, 26]. For instance, the loss of epigenetic factor KDM4B in BMSCs exacerbates skeletal ageing and osteoporosis by reducing bone formation and increasing marrow adiposity through elevated H3K9me3 [27]. Furthermore, the ablation of adiponectin‐expressing cells in the bone marrow results in a rapid and significant increase in systemic bone mass [8]. Consequently, we aim to identify new factors involved in the regulation of adipocyte differentiation in BMSCs, which may provide insights into the pathogenesis of osteoporosis and lead to targeted preventive and therapeutic strategies.

We have identified Foxk1 as a pivotal regulatory factor that facilitates the adipogenesis of BMSCs [20]. Although Foxks are generally involved in similar biological processes, mounting evidence suggests that the regulatory roles of Foxk1 and Foxk2 differ across various physiological and pathological contexts. Notably, Foxk1 and Foxk2 promote aerobic glycolysis by upregulating the enzymatic machinery necessary for this process, while simultaneously inhibiting further oxidation of pyruvate in the mitochondria through increased activity of pyruvate dehydrogenase kinases 1 and 4 [22]. They also exhibit reciprocal interactions with Foxo1, modulating its subcellular localisation between the cytoplasm and nucleus in response to insulin signalling [19]. However, in gastric cancer, their roles diverge: Foxk1 promotes epithelial‐mesenchymal transition, migration and invasion in vitro [28], while Foxk2 functions antagonistically [29]. Building on these findings, we further investigated the role of Foxk2 in adipogenesis.

Given that the differentiation process of adipocytes from bone marrow and WAT is remarkably similar, we first examined the expression levels of Foxk2 in peripheral adipose tissues. We found that Foxk2 expression was relatively low in iWAT and eWAT compared to skeletal muscle tissues in wild‐type C57BL/6J mice. However, Foxk2 was significantly upregulated in the iWAT and eWAT of obese db/db mice compared to non‐obese db/m mice (Figure 1). Next, we observed that Foxk2 was induced in primary BMSCs as well as in the cell lines C3H/10 T1/2 and ST2 during adipogenic differentiation, reaching its highest level of induction in the early stages (Figure 2). These findings indicate that the expression pattern of Foxk2 is similar to that of Foxk1 during adipogenic differentiation [20], suggesting that Foxk2 may also play a same role in this process.

We elucidated the role of Foxk2 in adipogenic differentiation through gain‐ and loss‐of‐function analyses. Foxk2 overexpression enhanced the proliferation of progenitor cell lines, including C3H/10 T1/2, ST2 cells, and primary BMSCs, while Foxk2 knockdown impaired their proliferation (Figure S1). Furthermore, Foxk2 overexpression promoted adipocyte differentiation and lipogenesis in C3H/10 T1/2 and ST2 cells (Figure 3), whereas its inhibition suppressed both adipocyte differentiation and lipogenesis in these cells and 3 T3‐L1 preadipocytes (Figure 4, Figure S3). Finally, the role of Foxk2 in adipogenesis was confirmed in primary BMSCs (Figure 5). These findings indicate that Foxk2 acts as a positive regulator of both adipogenesis and lipogenesis.

We next investigated the molecular mechanisms through which Foxk2 regulates adipogenesis. Fox TFs typically exert their regulatory effects by translocating to the nucleus. For instance, Foxi2 directly binds to the promoter of the AgRP gene, activating its transcription to enhance AgRP expression, which stimulates AgRP neuron activity, leading to increased food intake and reduced energy expenditure in mice [30]. Similarly, Foxp3 interacts with the Ikaros family TFs Ikzf1 and Ikzf3, forming a complex that competes with epigenetic co‐activators, such as p300, for binding at target gene loci. This interaction controls chromatin architecture, thereby repressing gene expression in Treg cells to limit autoimmunity and anti‐tumour immunity [31]. In our study, Foxk2 predominantly localised to the nucleus in C3H/10 T1/2 cells following adipogenic stimulation after its overexpression (Figure 6A–E). Previous studies have shown that both Foxk1 and Foxk2 regulate cellular metabolism, including glycolysis, lipid metabolism and hepatic fatty acid oxidation, in a manner dependent on the PI3K‐AKT and mTOR signalling pathways [19, 20, 21, 32]. Consistent with these findings, the PI3K inhibitor LY294002, Akt inhibitor MK‐2206 and mTOR inhibitor rapamycin significantly inhibited Foxk2 nuclear localisation after adipogenic treatment (Figure 6F–H). Furthermore, these inhibitors retained Foxk2 in the cytoplasm, resulting in impaired adipocyte differentiation (Figure 6I–L).

We then sought potential regulatory genes through which Foxk2 exerts its function. In the process of adipogenic differentiation, numerous studies have established that PPARγ is the master transcriptional regulator of adipogenesis. The mouse PPARγ gene expresses two isoforms: PPARγ2, whose expression is restricted to adipose tissues, and PPARγ1, which is expressed in a broader range of tissues. During adipogenesis, both PPARγ1 and PPARγ2 are markedly upregulated; however, their regulation differs due to the distinct locations of their promoters, with the PPARγ1 promoter situated far upstream of the PPARγ2 promoter. Additionally, members of the C/EBP family, including C/EBPα, C/EBPβ, C/EBPδ and C/EBPζ, are also key transcription factors that drive the differentiation of preadipocytes into mature adipocytes [33, 34, 35, 36]. Multiple Fox transcription factors (TFs) are involved in regulating C/EBP family members and PPARγ transcriptional activity, thus affecting adipogenesis. For instance, Foxa3 acts as a positive regulator of adipocyte differentiation by synergising with C/EBPβ or C/EBPδ to activate the PPARγ2 promoter [37]. Conversely, Foxo1 inhibits adipocyte differentiation and lipogenesis by binding to the PPARγ promoter and repressing PPARγ transcriptional activity, competitively preventing the formation of the functional PPARγ/RXR/DNA complex during terminal differentiation [38]. we previously demonstrated that Foxk1 directly binds to the PPARγ2 promoter and activates its transcription to promote adipogenesis [20]. In our current study, we found that Foxk2 can directly bind to the promoter regions of both Pparγ1 and Pparγ2. Furthermore, silencing PPARγ expression significantly abrogated the stimulatory effect of Foxk2 on adipogenesis (Figure 7). Given the reciprocal relationship between adipocyte and osteoblast differentiation from BMSCs [39, 40], we hypothesise that Foxk2 may also play a crucial role in osteogenesis. Future studies will focus on investigating its function in bone formation.

In summary, we found that Foxk2 expression is upregulated in the iWAT and eWAT of obese mice and is enhanced in primary BMSCs and progenitor cell lines following adipogenic stimulation. This study is the first to demonstrate that mouse Foxk2 plays a positive role in adipogenesis and lipogenesis in vitro. Additionally, Foxk1 and Foxk2 appear to exhibit functional redundancy in this process, as co‐overexpression or co‐knockdown of these genes did not enhance or diminish each other's effects on adipogenesis. Mechanistically, our findings suggest that adipogenic stimulation induces the nuclear translocation of Foxk2, which subsequently binds directly to the PPARγ1 and PPARγ2 promoters, increasing transcriptional activity and promoting adipocyte differentiation (Figure 7L). Our study proposes that Foxk2 may serve as a potential therapeutic target for ageing‐associated or oestrogen loss‐induced BMAT accumulation and osteoporosis. However, silencing PPARγ1 and PPARγ2 expression did not fully abrogate the stimulatory effect of Foxk2 on adipogenesis, indicating the involvement of additional Foxk2 target genes that remain to be identified. Moreover, as the current findings are primarily based on in vitro experiments, further in vivo studies are required to elucidate the physiological role of Foxk2.

## Author Contributions

**Shan Zhang:** investigation (lead). **Yanru You:** investigation (equal), validation (lead). **Ran Li:** investigation (supporting), validation (supporting). **Mingcong Li:** investigation (supporting), validation (supporting). **Yachong Li:** investigation (supporting), validation (supporting). **Hairui Yuan:** project administration (equal). **Jie Zhou:** project administration (equal). **Ruonan Zhen:** project administration (equal). **Ying Liu:** supervision (equal). **Baoli Wang:** supervision (equal), writing – review and editing (equal). **Endong Zhu:** conceptualization (lead), data curation (lead), supervision (lead), writing – original draft (lead), writing – review and editing (lead).

## Ethics Statement

All animal experiments were carried out following the National Standard of Animal Care and Use Procedures and were approved by the Animal Ethics Committee of Tianjin Medical University Chu Hsien‐I Memorial Hospital (approval number: DXBYY‐IACUC‐2022036).

## Conflicts of Interest

The authors declare no conflicts of interest.

## Supporting information


Figure S1.
Figure S2.
Figure S3.

## Data Availability

The data that support the findings of this study are available from the corresponding author upon reasonable request.
