# Supplementary material for: Foxk2 Enhances Adipogenic Differentiation by Relying on the Transcriptional Activation of Peroxisome Proliferator‐Activated Receptor Gamma
Source: J Cell Mol Med. 2025 Jan 9;29(1):e70332. doi: 10.1111/jcmm.70332 (PMC11717668; doi:10.1111/jcmm.70332)
Supplement: Supplementary file 1 — Figure S1. Figure S2. Figure S3. [file JCMM-29-e70332-s001.doc]

**Supplemental Figures and Legends**


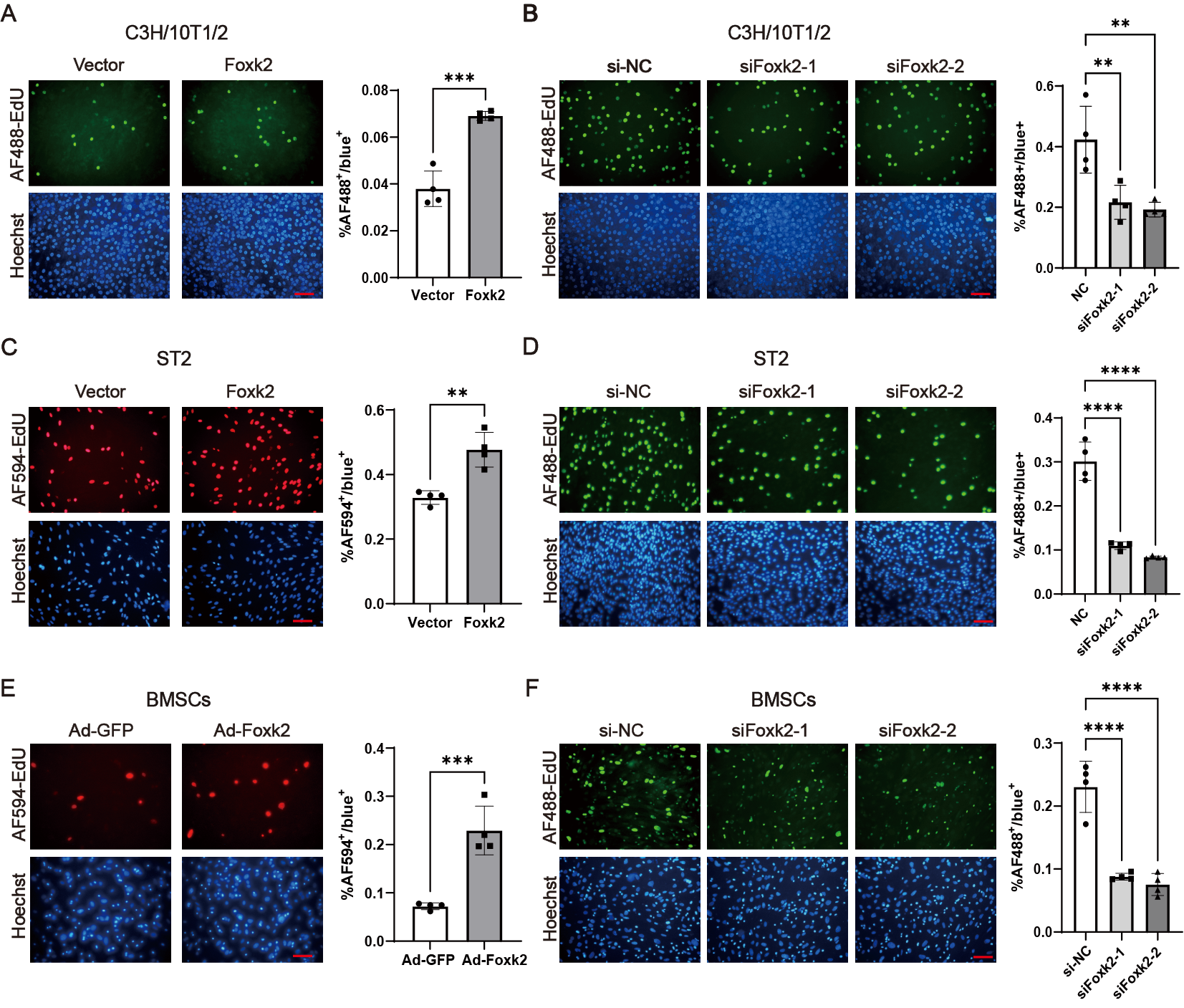


**Figure S1. The effect of Foxk2 on cell proliferation**

EdU staining assay was perform to examine the role of Foxk2 in proliferation of C3H/10T1/2 (A, B) and ST2 cells (C, D) 2 days after transfection with indicated coding construct or siRNAs, or in primary BMSCs infected with indicated adenovirus or transfection with indicated siRNAs (E, F). The percentage of EdU-positive cells were analyzed by ImageJ software. Scale bars, 100 μm. The values are mean±S.D., P < 0.05 (*), P < 0.01 (**), P < 0.001 (***), P < 0. 0001 (****), presented relative to control group (A two-tailed student’s t test was applied between two groups, and ANOVA followed by Tukey’s test was applied between more than two groups).


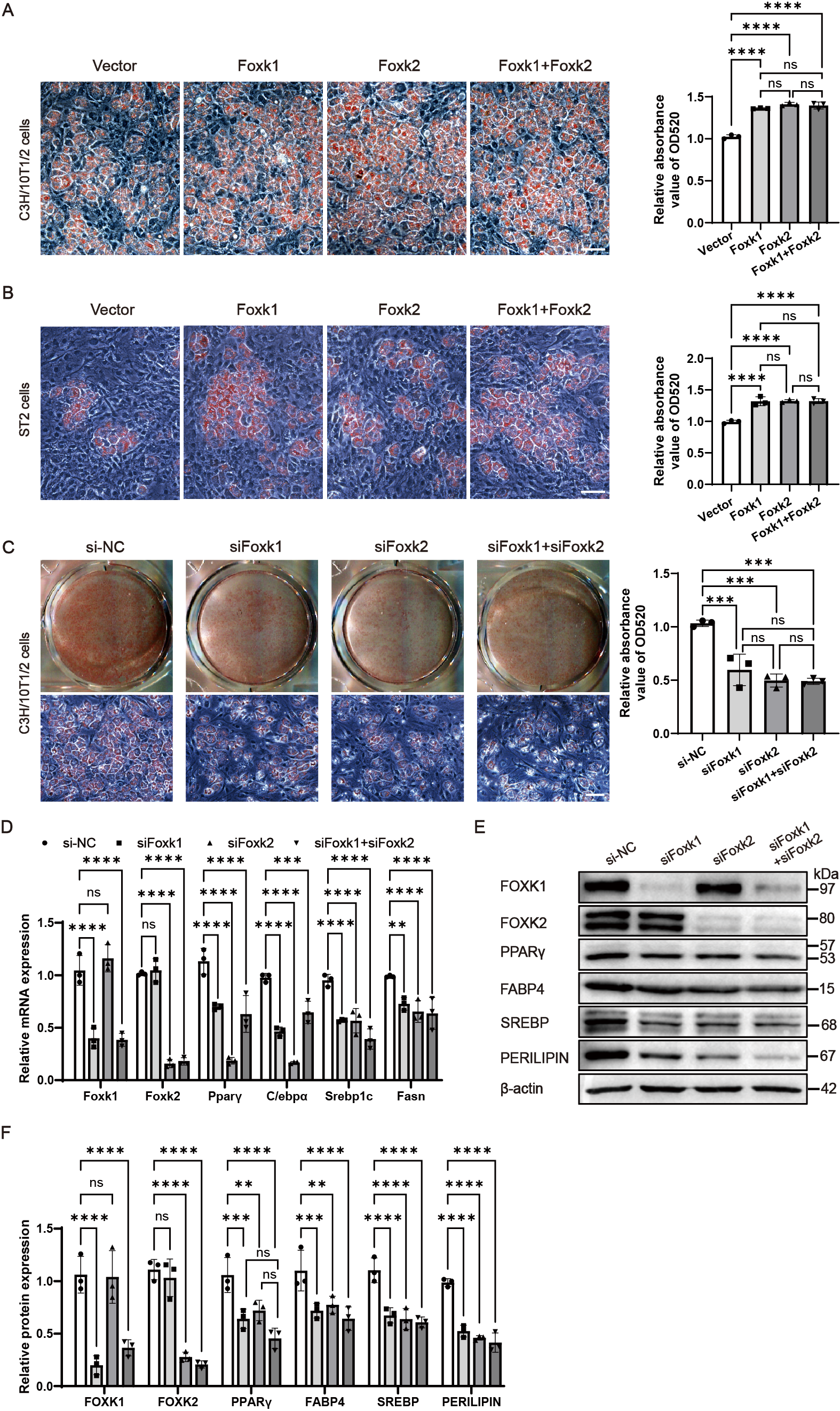


**Figure S2. The combined Foxk1 did not further enhance the effect of Foxk2 on adipogenic differentiation and lipogenesis**

(**A**) C3H/10T1/2 or (**B**) ST2 cells were transfected with vector control, Foxk1, Foxk2, or a combination of Foxk1 and Foxk2 expression constructs, followed by adipogenic treatment for 5 days. Adipocyte differentiation was assessed by Oil Red O staining, and representative images are shown. Scale bars represent 100 μm. The Oil Red O stain was extracted with isopropanol and quantified by measuring absorbance at OD520. (**C**) C3H/10T1/2 cells were transfection with si-NC, Foxk1 siRNA (siFoxk1), siFoxk2 (siFoxk2-1), or a combination of Foxk1 and Foxk2 siRNA (siFoxk1+ siFoxk2). Cells were then cultured in AIM for 5 days, followed by Oil Red O staining to assess adipocyte differentiation, with representative images shown (scale bars, 100 μm). Oil Red O was extracted with isopropanol and measured at OD520. RT-qPCR (**D**) and western blotting (**E**) were performed to test the expression of the adipocyte-specific genes C/EBPα, PPARγ and FABP4, and lipogenesis marker genes Srebp1c, Fasn and Perilipin. The blots were further quantified with grayscale **(F)**. All micrographs were taken using a light microscope. β-actin served as an internal control. The values are mean±S.D., P < 0.05 (*), P < 0.01 (**), P < 0.001 (***), P < 0. 0001 (****), presented relative to the control group (ANOVA followed by Tukey’s test was applied among three groups).


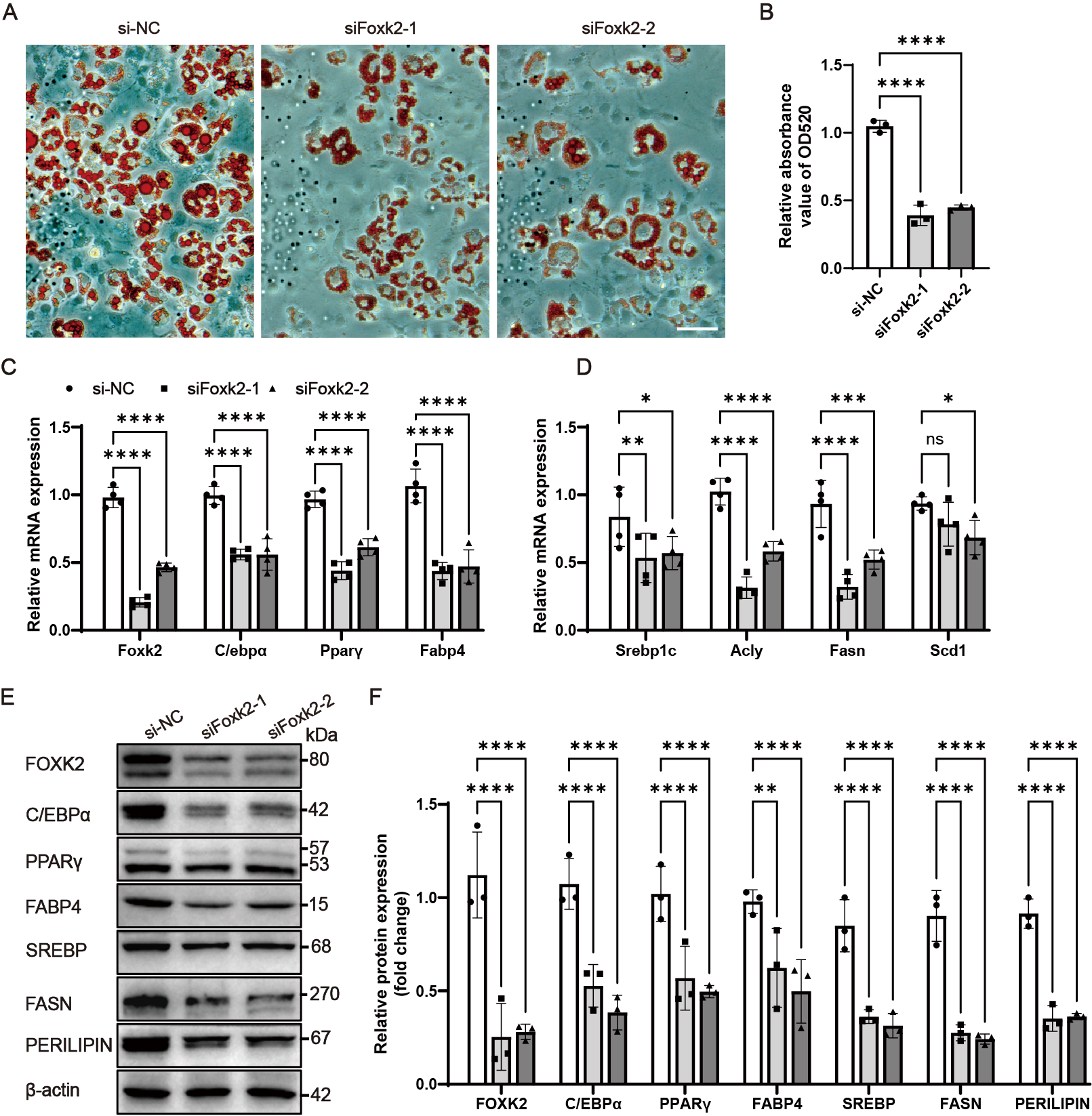


**Figure S3. Reduced expression of Foxk2 impaired adipocyte differentiation and lipogenesis in 3T3-L1 preadipocytes**

(**A**) Oil-red O staining was performed in adipocyte-differentiated 3T3-L1 cells (Scale bars, 100μm), and the stain extracted with isopropanol was measured at 520 nm by spectrophotometry (**B**). The mRNA (**C, D**) and protein (**E**) levels of adipogenic and lipogenic factors were measured 3 days after adipogenic treatment. Western blot bands were quantified by grayscale analysis (**F**). β-actin served as an internal control. Data are presented as mean ± S.D. Statistical significance was determined using a two-tailed Student’s t test, with significance indicated as *P < 0.05, **P < 0.01, ***P < 0.001, and ****P < 0.0001 compared to the control group.
